# Late Depression and Senescence-Associated Secretory Phenotype (SASP)

**DOI:** 10.1192/j.eurpsy.2025.1756

**Published:** 2025-08-26

**Authors:** A. Sidenkova

**Affiliations:** Psychiatry, Ural State Medical University, Ekaterinburg, Russian Federation

## Abstract

**Introduction:**

Neurobiological mechanisms of late depression include abnormal regulation and interaction of multiple biological processes, as well as abnormalities of brain structures and organs.

**Objectives:**

literature analysis

**Methods:**

general scientific method

**Results:**

The results of the study show that the SASP index as an integral indicator of changes in proteins associated with the cellular aging secretory phenotype is significantly higher in patients with late depression. A high SASP index correlates with older age and age-related diseases. A high SASP index correlates with low cognitive function (low executive function, information processing speed), and high gray matter atrophy. According to the authors, this indicates a connection between late depression and the molecular picture caused by structural and phenotypes of brain aging. Postmortem studies of patients with late depression revealed a coincidence of abnormal gene expression with genes whose expression is regulated by age. Proteomic studies show that late-onset depression associated with cognitive impairment is associated with abnormal regulation of biological pathways related to immune inflammation control, proteostasis, cellular communication and signaling, and oxidative stress. These biological pathways were downregulated during aging, suggesting that depression is associated with lifelong biological abnormalities, limitations with aging. Studies have shown that some of the biomarkers associated with inflammation, growth factors, and cell surface proteins are abnormally regulated in late-onset depression (e.g., elevated sTNFR2 levels) or are associated with certain clinical characteristics such as cognitive impairment (e.g., angiogenin, IGFBP-6). The biological effects of SASP proteins are mediated by p53, p38MAPK, and NFκB signaling pathways. The SASP index correlates with poor executive function and information processing speed. Patients with late-onset depression associated with cognitive decline have been shown to have profound neurobiological abnormalities and significantly reduced BDNF levels, which is consistent with an enhanced molecular profile of aging. SASP proteins can be produced and secreted by senescent glial cells and activate neuronal intracellular cascades associated with nutrition, metabolic control, cell growth, and apoptosis.

**Conclusions:**

Key to understanding the neurobiology of late-onset depression is how it interacts with brain and systemic biological changes associated with aging.

**Disclosure of Interest:**

None Declared

